# Long-term clinical follow-up of intracranial aneurysms treated with flow diverter devices: a bicentric retrospective study

**DOI:** 10.1186/s41016-026-00429-7

**Published:** 2026-03-20

**Authors:** Mahmoud Moubark, Alessandra Biondi, Moustafa Othman, Michael Findler, Giovanni Vitale, Francis Turjman

**Affiliations:** 1 Assiut University, Assiut, Egypt; 2 Centre Hospitalier Universitaire de Besançon, Besançon, France; 3 Department of Neurology, Rabin Medical Center—Beilinson Hospital, Petah Tikva, Israel; 4 Pierre Wertheimer Hospital in Lyon, Lyon, France

## Abstract

**Background:**

Flow diverter devices (FDDs) have revolutionized the treatment of intracranial aneurysms. However, comprehensive long-term outcome data across multiple device platforms remain limited, with most prior studies focusing exclusively on Pipeline embolization device (PED) outcomes. This study reports extensive long-term clinical follow-up of patients treated with various types of FDDs, providing critical safety and efficacy data for contemporary practice.

**Methods:**

We conducted a retrospective analysis of intracranial aneurysms treated with FDDs from February 2011 to July 2016 in two tertiary care centers in France. The study included 209 patients with 216 cerebral aneurysms (91.2% anterior circulation, 8.8% posterior circulation; 66.2% unruptured, 31.0% recanalized, 2.8% ruptured). The duration of clinical and angiographic follow-up ranged from 24 to 156 months. Kaplan–Meier analysis performed for complication-free survival and aneurysm occlusion durability. Multivariate analysis showed risk factors for delayed complications.

**Results:**

Long-term follow-up data were available for 202/209 (96.7%) patients. The early permanent morbidity and mortality rates were 3.8% and 1.4%, respectively. Between 12 and 18 months, procedure-related complications occurred in 5 of 202 patients (2.5%): two ischemic events, one delayed rupture, one silent intrastent stenosis, and one case of seizures following antiplatelet withdrawal. Only three patients (1.5%) were symptomatic. At the 24-month follow-up, complete aneurysm occlusion achieved in 81.0% of the patients, with adequate occlusion in 93.3%. Kaplan–Meier analysis revealed 97.5% complication-free survival at 18 months, with sustained durability thereafter. Multiple FDDs were the only significant independent predictor of delayed complications (OR: 7.066, 95% CI: 1.049–47.606, *P* < 0.045). No procedure-related complications or deaths occurred after 18 months.

**Conclusion:**

Beyond 18 months of follow-up, FDD treatment for intracranial aneurysms proves exceptional safety with no delayed complications, regardless of device type. However, the findings of this study also show 18 months as a critical threshold for complication risk assessment, a key implication that clinicians and researchers need to be aware of. The study also underscores the need for modified management strategies when multiple overlapping FDDs are deployed, which significantly increases the risk of delayed complications.

## Background

Endovascular treatment (EVT) has emerged as the first-line therapy for most intracranial aneurysms following the landmark International Subarachnoid Aneurysm Trial (ISAT), which demonstrated superior outcomes compared with surgical clipping [1]. Among the various endovascular technologies, flow diverter devices (FDDs) represent a paradigm shift in aneurysm treatment, fundamentally altering the therapeutic approach from intrasaccular occlusion to parent vessel reconstruction [2].

FDDs function by modifying the hemodynamic interface between the parent artery and the aneurysm sac. Following deployment, the high-density mesh promotes intrasaccular thrombosis while facilitating neointimal growth, which reconstructs the parent vessel wall. This process effectively eliminates the aneurysm-parent vessel interface while preserving perforator and branch vessel patency [3]. This technology has proven particularly valuable for treating large or giant aneurysms, complex morphology lesions, and recurrent aneurysms that pose high risks for conventional coiling approaches [4].

Despite widespread adoption and numerous studies evaluating FDD efficacy, comprehensive long-term clinical outcome data remain surprisingly limited. Most literature focuses predominantly on short-term results or single-device experiences, particularly with the Pipeline embolization device (PED) [5, 6]. This narrow focus creates significant knowledge gaps regarding long-term safety profiles across different FDD platforms. This limits our understanding of delayed complication patterns that may inform clinical decision-making and patient counseling.

The current study addresses these critical knowledge gaps by reporting the most extensive long-term clinical follow-up data available across multiple FDD platforms. Unlike earlier PED-centric studies, our multidevice analysis provides insights into universal safety patterns and risk factors that transcend individual device characteristics. Furthermore, our extended follow-up period (up to 156 months) offers unprecedented insight into the temporal evolution of complications, revealing critical thresholds that have important implications for patient management and surveillance strategies.

## Methods

### Study design and population

We performed a retrospective analysis of consecutive patients with intracranial aneurysms treated with flow diverter devices between February 2011 and July 2016 at two tertiary care centers in France: Pierre Wertheimer Hospital, Lyon, and Besancon University Hospital.

This retrospective multicenter study was conducted at Pierre Wertheimer Hospital, Lyon, France, and Centre Hospitalier Universitaire de Besançon, Besançon, France. The study protocol was reviewed and approved by the Scientific and Ethical Committee of Hospices Civils de Lyon (IRB No. 00013204) and by the Comité de Protection des Personnes Est II, Besançon, France (Approval No. 2011-A01013-38).

Given the retrospective nature of the study and the use of fully anonymized clinical and imaging data, the requirement for written informed consent was waived by the respective institutional review boards. All procedures were conducted in accordance with the ethical standards of the institutional and national research committees and with the principles of the Declaration of Helsinki and its later amendments.

Data handling and processing were performed in compliance with the General Data Protection Regulation (GDPR) (EU 2016/679) and applicable French data protection regulations.

### Inclusion and exclusion criteria

The inclusion criteria were as follows:

- Large or giant unruptured intradural saccular or fusiform aneurysms (> 10 mm)
- Wide-neck aneurysms (dome-to-neck ratio < 2 or neck width > 4 mm) at operator discretion
- Small aneurysms (< 3 mm) deemed unsuitable for conventional coiling techniques
- Recurrent aneurysms following previous treatment modalities

The exclusion criteria were as follows:

- Treatment before February 2011 (to eliminate first-generation device effects and learning curve bias)
- Treatment after July 2016 (to ensure adequate long-term follow-up duration)
- Parent artery stenosis 50% at the intended FDD placement site
- Inability to tolerate dual antiplatelet therapy

### Treatment protocol

All procedures performed under general anesthesia via a standardized triaxial approach via femoral artery access, with one patient requiring brachial access due to challenging aortic arch anatomy. FDD deployment utilized proper microcatheters: 0.027 inches for Pipeline, Surpass, p64, and FRED devices and 0.023 inches for SILK devices. Device choice was based on aneurysm characteristics, parent vessel anatomy, and operator preference.

### Antiplatelet management

Patients received standardized dual antiplatelet therapy consisting of clopidogrel (75 mg daily) and aspirin (150 mg daily), which was started 5–7 days before the intervention. Platelet function testing performed via the VerifyNow P2Y12 assay (Accumetrics, San Diego, CA) for clopidogrel responsiveness and the VerifyNow Aspirin assay for aspirin responsiveness before intervention.

Dual antiplatelet therapy continued for 3–6 months post-procedure, depending on the clinical context and presence of intimal hyperplasia, followed by lifelong aspirin monotherapy. Protocol modifications addressed clopidogrel nonresponders (switching to abciximab or double-dose clopidogrel) and hyper-responders (half-dose clopidogrel).

### Follow-up protocol

Angiographic assessment: Digital subtraction angiography (DSA) performed at 3–6 months and 12 months post-procedure, with other DSA between 24 and 48 months. Aneurysm occlusion evaluated via the O’Kelly–Marotta (OKM) [7] and Cekirge [8] grading scales. Extended radiological evaluation (5–7.5 years) utilizes magnetic resonance angiography (MRA) or computed tomography angiography (CTA) based on occlusion progression.

Clinical assessment: Clinical follow-up occurred at 3–6 months, 12 months, and long-term intervals (24–156 months). Outcomes assessed via the modified Rankin scale (mRS) by independent neurologists not involved in treatment. Additional follow-up conducted via structured telephone interviews from August to September 2018 to verify the clinical status of the patients.

### Statistical analysis

Categorical variables presented as numbers and percentages; continuous variables are presented as medians ± interquartile ranges and means ± standard deviations. Kaplan–Meier survival analysis performed for complication-free survival and aneurysm occlusion durability. Univariate analysis identified potential predictors of complications beyond 12 months, with variables with *P* < 0.20 included in multivariate conditional logistic regression analysis. Statistical significance was set at *P* < 0.05. Analyses conducted via IBM SPSS version 28.0 (IBM Corp., Armonk, NY, USA).

The variables analyzed in the multivariate analysis included the following:

- Aneurysm characteristics (complex size, wide neck, complex configuration, intraluminal thrombus, incorporated artery, and same-segment aneurysm)
- Treatment variables (number of FDDs, device type, additional coiling, previous treatment)
- Procedural complications

## Results

### Patient demographics and aneurysm characteristics (Table 1)

**Table 1:** Baseline demographics and aneurysm characteristics

Characteristic	Category	*N* (%)
Total aneurysms		216 (100.0)
Aneurysm location	Anterior circulation	197 (91.2)
	Posterior circulation	19 (8.8)
Aneurysm size	Small (< 8 mm)	99 (45.8)
	Medium (8–12 mm)	59 (27.3)
	Large (13–24 mm)	47 (21.8)
	Giant (≥ 25 mm)	11 (5.1)
Aneurysm shape	Saccular	167 (77.3)
	Fusiform	17 (7.9)
	Dissecting	30 (13.9)
	Blister	2 (0.9)
Neck size*	< 4 mm	63 (37.7)
	≥ 4 mm	104 (62.3)
Dome-to-neck ratio*	≤ 1.5	86 (51.5)
	> 1.5	81 (48.5)
Other features	Thrombosed aneurysm	9 (4.2)
	Incorporated vessels	51 (23.6)
	Additional aneurysm on same segment	28 (12.9)

Neck size and dome-to-neck ratio are reported for saccular aneurysms only (*n* = 167)

The cohort comprised 209 patients (mean age 54.2 ± 12.8 years, 78.5% female) with 216 target aneurysms. The aneurysm locations included the internal carotid artery (ICA) (142, 65.7%), anterior cerebral artery (ACA) (32, 14.8%), middle cerebral artery (MCA) (23, 10.6%), and posterior circulation (19, 8.8%). The mean aneurysm size was 8.4 ± 5.2 mm, with 143 (66.2%) unruptured, 67 (31.0%) recanalized after previous treatment, and 6 (2.8%) ruptured aneurysms.

### Device deployment and technical outcomes

Successful FDD deployment achieved in 212/216 procedures (98.1%), with deployment failure in 4 procedures (1.9%). A total of 258 FDDs deployed across 216 procedures: a single FDD in 181 procedures (83.8%), two FDDs in 31 procedures (14.3%), three FDDs in 2 procedures (0.9%), and four or five FDDs in one procedure each (0.5%). The device distributions included Pipeline 98 (38.0%), SILK 89 (34.5%), Surpass 41 (15.9%), p64 18 (7.0%), and FRED 12 (4.7%).

### Early complications

Periprocedural complications occurred in 9/209 patients (4.3%). Parent artery occlusion developed in 5 patients (2.4%), involving the carotid cavernous (*n* = 1), posterior communicating artery (*n* = 1), A1–A2 junction (*n* = 1), anterior choroidal artery (*n* = 1), and carotid terminus–M1 junction (*n* = 1) locations. Five patients (2.4%) were symptomatic, with outcomes including two patients who achieved an mRS score of zero at discharge, two who experienced permanent morbidity (mRS score of 3), and one who died (0.47%) due to bleeding following anterior cerebral artery perforation.

Postprocedural complications within 30 days affected 13 patients (6.2%) (Table 2):

- Two transient ischemic attacks
- Three ischemic events
- Two parenchymal hemorrhages
- One RT Terson syndrome of the eye
- One fatal aneurysm rupture
- One case of fatal brainstem compression
- One incomplete Horner syndrome patient
- One patient with blurred vision and central scotoma
- One case of confusion

**Table 2:** Clinical and procedural details of patients with complications < 1 year

Case no	Aneurysm location	Number of FDDs	FDD type	Complication	Time to event (days)	mRS base	Last mRS
30	LT ophthalmic	1	PED	Hemorrhage	11	0	2
52	LT ophthalmic	1	Surpass	Aneurysm rupture	5	0	6
112	RT MCA	1	P64	Ischemia	90	0	0
113	ACOM	1	Surpass	Ischemia	1 (< 24 h)	0	1
119	RT PCOM	1	PED	Blurred vision	1 (< 24 h)	2	2
141	LT ICA	1	SILK	Horner syndrome	1 (< 24 h)	0	0
171	Basilar	5	PED	Brain stem compression	17	3	6
177	RT PCOM	1	PED	Ischemia	1 (< 24 h)	0	3
185	RT vertebral (V4)	1	Surpass	Hemorrhage	90	0	3
188	ACOM	1	PED	RT Terson syndrome of the eye	4	0	0
190	RT PCOM	1	PED	TIA	2	2	0
192*	RT ICA	2	P64	Confusion	1 (< 24 h)	1	6
195	RT PCOM	2	PED	TIA	2	1	2
196**	LT PCOM	2	PED	Hemorrhage	18	0	4
196**	LT PCOM	2	PED	Ischemia	155	0	4
205	RT ICA	1	PED	Ischemia	20	0	1

*Abbreviations: FDD* flow diverter device, *mRS* modified Rankin scale, *LT* left, *RT* right, *MCA* middle cerebral artery, *ACOM* anterior communicating artery, *PCOM* posterior communicating artery, *ICA* internal carotid artery, *PED* Pipeline embolization device, *TIA* transient ischemic attack

*Case 192 non-neurological death

**Case 196 both hemorrhagic and ischemic complications occurred in different time periods

Access site complications occurred in 8/216 procedures (3.7%), including six subcutaneous hematomas, one retroperitoneal hematoma, and one femoral arteriovenous fistula, all of which were managed conservatively.

### Short-term outcomes (1–12 months) (Table 2)

Short-term complications (1–6 months) occurred in 3 patients (1.4%): one parenchymal hemorrhage at 3 months and two ischemic events at 3 and 5 months. Four deaths unrelated to FDD treatment occurred between 9 and 14 months (pancreatic disease, cardiac stroke, trauma, and contralateral cerebral stroke).

### Midterm outcomes (12–18 months) (Table 3)

**Table 3:** Clinical and procedural details of patients with midterm complications (12–18 months)

Characteristic	Patient #10	Patient #29	Patient #50	Patient #65	Patient #183
Aneurysm location	LT PCOM	LT ophthalmic	RT MCA	RT ICA	RT PCOM
Presentation	Incidental	Recurrent	Headache	CN palsy	Trigeminal neuralgia
Number of FDDs	2	1	1	2	2
FDD type	PED	PED	PED	SILK	PED
Complication	Ischemia	Seizures	Silent ISS	Ischemia	Delayed rupture
Time to event (days)	370	381	369	367	369
Clinical implication	Hemiplegia	Seizures	None	None	Dysarthria, confusion
Final outcome	Partially improved	Improved	No deficit	No deficit	Partially improved
mRS at discharge	0	0	0	1	1
Final mRS	2	0	0	1	1

*Abbreviations: LT* left, *PCOM* posterior communicating artery, *MCA* middle cerebral artery, *ICA* internal carotid artery, *CN* cranial nerve, *FDD* flow diverter device, *PED* Pipeline embolization device, *ISS* intrastent stenosis

**Fig. 1:**
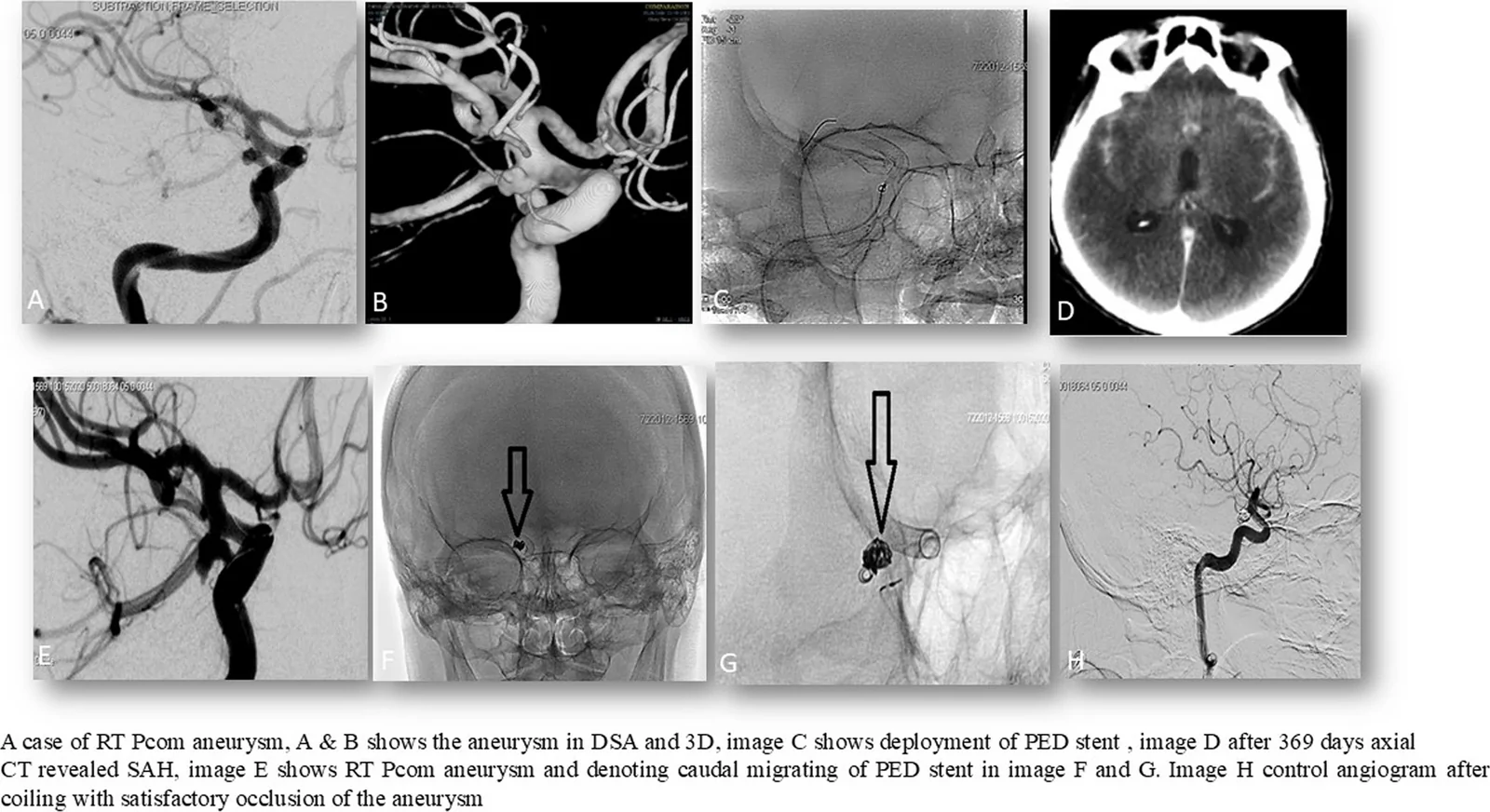
A 52-year-old male patient with an RT PCOM aneurysm (4.7 × 6.2 × 3.6 mm) complaining of trigeminalneuralgia with no comorbidities was treated with two PED stents (3.5 × 18 mm and 3.5 × 20 mm) with incomplete stent coverage. At the 1-year follow-up, proximal stent migration, increased aneurysm size, and rupture occurred 3 days after coiling. Follow-up after retreatment revealed complete occlusion of theaneurysm (mRS = 1) at 36 months (dysarthria after rupture)

Between 12 and 18 months, procedure-related complications occurred in 5/202 patients (2.5%):

- Ischemic complications (*n* = 2): Both patients treated with multiple overlapping FDDs. One patient experienced transient weakness attributed to in-stent stenosis progression, whereas another developed stroke symptoms potentially related to device misalignment requiring in-stent angioplasty.
- Delayed aneurysm rupture (*n* = 1): A 52-year-old patient with a small posterior communicating artery aneurysm (aspect ratio 1.72) experienced delayed rupture at 12 months. Angiographic analysis revealed proximal FDD migration (Fig. 1), potentially redirecting flow into the aneurysm and preventing complete thrombosis.
- Silent intrastent stenosis (*n* = 1): Asymptomatic stenosis developed in a patient with a large dissecting MCA aneurysm treated with the Pipeline device. The patient remained neurologically intact with conservative management.
- Antiplatelet withdrawal-related seizures (*n* = 1): A unique case involved a patient with an ophthalmic aneurysm who developed epileptic seizures 2 weeks after aspirin cessation at 1-year post-treatment. Seizures resolve upon aspirin resumption, although the causal relationship remains speculative and requires further investigation. Among these five patients with complications, only 3 (1.5%) were clinically symptomatic.

### Long-term outcomes (> 18 months)

Long-term clinical follow-up data were available for 202/209 patients (96.7%), with follow-up durations ranging from 24 to 156 months (median 48 months). Notably, no procedure-related complications or deaths occurred after 18 months. The mRS score remained unchanged in 201 patients (99.5%) and improved in one patient (0.5%) between 18 months and the final follow-up.

### Kaplan–Meier survival analysis

K–M analysis revealed excellent long-term outcomes:

- Complication-free survival rates were 97.5% at 18 months, 97.5% at 5 years, and 97.5% at the final follow-up.
- Aneurysm occlusion durability: 93.3% adequate occlusion was maintained throughout the follow-up period.
- Overall survival: 96.7% at the final follow-up (procedure-related mortality 1.4%).

### Angiographic outcomes

Complete angiographic follow-up data were available for 200/209 patients (95.7%). At the final assessment, complete aneurysm occlusion was observed in 171/211 aneurysms (81.0%), with adequate occlusion in 197/211 (93.3%). Eight target aneurysms (3.7%) needed retreatment: seven with other FDD and one with coiling.

Among the 28 adjacent aneurysms incidentally covered by the same stent, 15 (53.6%) achieved occlusion at the 1-year follow-up. Four aneurysms shown neck recanalization at 2–3 years, and three aneurysms (10.7%) showed persistent filling, with one requiring coil treatment at 42 months.

### Risk factor analysis (Table 4)

**Table 4:** Risk factor analysis for delayed complications (> 12 months)

Variable	Univariable analysis		Multivariable analysis	
	HR (95% CI)	*P* value	HR (95% CI)	*P* value
Complex size	0.814 (0.133–4.972)	0.824	–	–
Wide neck	0.712 (0.117–0.351)	0.713	–	–
Complex configuration	0.454 (0.074–2.793)	0.394	–	–
Intraluminal thrombus	6.340 (0.635–63.424)	0.116	2.821 (0.243–32.794)	0.407
Incorporated artery	1.242 (0.136–11.371)	0.848	–	–
Previous treatment	0.549 (0.060–5.004)	0.595	–	–
**Multiple FDD deployment**	**8.341 (1.348–52.218)**	**0.023**	**7.066 (1.049–47.606)**	**0.045**

Variables with *P* < 0.20 in the univariable analysis were included in the multivariable model. Significant findings are shown in bold

*Abbreviations: CI* confidential interval, *FDD* flow diverter device, *HR* hazard ratio

Univariate analysis identified several potential risk factors for delayed complications (> 12 months):

- Multiple FDD deployment (*P* = 0.032)
- Aneurysm aspect ratio > 1.6 (*P* = 0.089)
- Presence of intrasaccular thrombus (*P* = 0.116)
- Current smoking status (*P* = 0.158)
- Previous failed treatment (*P* = 0.187)

Multivariate analysis found multiple FDD deployments as the only significant independent predictor of delayed complications (odds ratio: 7.066; 95% CI: 1.049–47.606; *P* = 0.045). Patients treated with multiple overlapping devices demonstrated a sevenfold increased risk of experiencing delayed complications compared with patients receiving single-device treatment.

### Final clinical outcomes

**Fig. 2:**
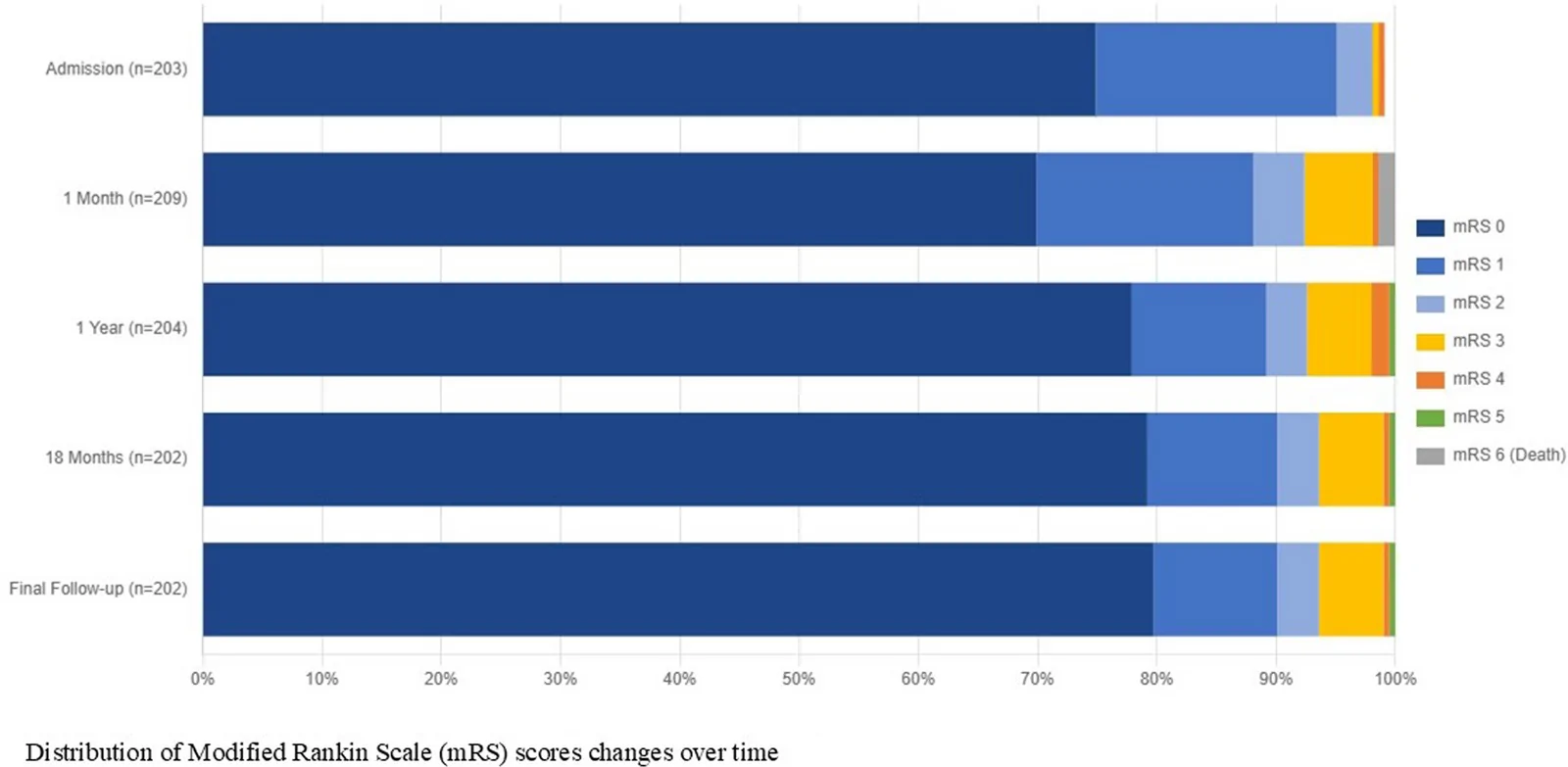
Distribution of modified Rankin scale (mRS) scores over time. The stacked bar chart illustrates the proportional distribution of functional outcomes, as measured by the modified Rankin scale (mRS), at different time points from admission to the final long-term follow-up. A clear trend towards improved functional outcomes was observed, with an increasing proportion of patients achieving mRS 0–1 over the follow-up period. Note: The 7 total deaths occurred by 18 months. Patients who died were not included in subsequent follow-up counts

At the final follow-up, FDD-related mortality remained at 1.4% (3/209), with an overall mortality of 3.3% (7/209). Device-related major stroke or death rates were 2.8% at 30 days, 0.48% at 1 year, and 0% after 18 months. The device-related minor stroke rates at the corresponding time points were 2.4%, 0%, and 1.0%, respectively (Fig. 2).

## Discussion

### Long-term safety profile across multiple FDD platforms

This study provides the most comprehensive long-term safety data available for FDD treatment across multiple device platforms. Unlike earlier studies that focused predominantly on Pipeline device outcomes, our multidevice analysis demonstrated that excellent long-term safety profiles are universal across different FDD technologies, including Pipeline, SILK, Surpass, p64, and FRED devices. This finding has important clinical implications, suggesting that long-term safety considerations should focus on patient and procedural factors rather than specific device characteristics.

Our results demonstrate that FDD-related complications become exceptionally rare beyond 18 months, with zero procedure-related complications or deaths occurring after this critical threshold. This temporal pattern provides definitive evidence for risk counseling and establishes 18 months as a landmark for complication risk assessment. The Kaplan–Meier analysis confirmed the sustained safety, with 97.5% complication-free survival maintained throughout the entire follow-up period.

### Risk stratification and clinical decision-making

The identification of multiple overlapping FDDs as the sole independent predictor of delayed complications (OR: 7.066) represents a crucial finding for clinical practice. This sevenfold increased risk provides quantitative evidence for treatment planning, suggesting that single-device strategies should be pursued whenever technically possible. When multiple devices are necessary, patients need enhanced surveillance and potentially extended antiplatelet therapy protocols.

The mechanisms underlying increased risk with multiple FDDs likely include increased intimal proliferation, altered flow dynamics, and increased thrombogenic potential at device overlap zones. These findings complement the literature suggesting that device complexity correlates with complication risk [9–11], but our study provides the first quantitative long-term risk assessment across multiple platforms.

### Novel clinical observations

Our study documents several clinically significant observations that expand the understanding of FDD-related complications:

- Device migration and delayed rupture: Delayed aneurysm rupture cases with documented FDD migration provide critical mechanistic insights. The combination of a high aspect ratio (1.72) and proximal device migration likely creates unfavorable hemodynamics, preventing thrombosis while maintaining high intrasaccular pressure [12, 13]. This observation emphasizes the importance of optimal device positioning and suggests that small aneurysms with high aspect ratios may require particular attention during long-term follow-up.
- Antiplatelet withdrawal phenomena: Seizures following aspirin withdrawal represent a novel observation requiring careful interpretation. While the temporal relationship suggests a potential association, the mechanism remains speculative. This anecdotal finding emphasizes the need for cautious antiplatelet discontinuation protocols and careful monitoring during transition periods.
- Silent intrastent stenosis: A single case of delayed, significant intrastent stenosis in a dissecting aneurysm highlights the importance of underlying vessel pathology in the development of complications. Most cases of intrastent stenosis are asymptomatic and self-resolving [14], but our observations suggest that dissecting lesions may require enhanced surveillance.

### Comparison with existing literature

Our long-term complication rates compare favorably with those of recent meta-analyses of FDD outcomes [15]. However, our study provides several unique contributions:

1. Extended follow-up duration: The 156-month maximum follow-up exceeds that of most published series, offering unprecedented insight into very long-term outcomes.
2. Multi-device platform analysis: Unlike PED-focused studies, our comprehensive device analysis proves universal safety principles across FDD technologies.
3. Quantitative risk assessment: The identification and quantification of multiple device risks provide actionable clinical guidance that is not available in earlier studies.
4. Temporal complication analysis: The identification of 18 months as a critical safety threshold offers new insights into complication timing and risk evolution.

### Clinical implications and future directions

These findings have several important clinical implications:

- Patient selection: The excellent long-term safety profile supports FDD use for proper indications, with particular consideration of device number requirements during treatment planning.
- Surveillance protocols: The absence of complications beyond 18 months suggests that intensive surveillance may be reduced after this threshold, although continued monitoring is still necessary for aneurysm occlusion assessment.
- Antiplatelet management: The single case of withdrawal-related seizures suggests that antiplatelet discontinuation protocols should include careful neurological monitoring, although this observation requires validation in larger series.
- Device strategy: The quantified risk of multiple devices supports single-device approaches when technically possible, with enhanced protocols for complex cases requiring multiple devices.

### Study limitations

Several limitations warrant acknowledgment:

- Temporal considerations: The study period (2011–2016) ensures adequate follow-up but precedes recent device improvements and technical advances. However, the fundamental hemodynamic principles and complication patterns remain relevant to contemporary practice.
- Retrospective design: Inherent limitations include potential selection bias and incomplete data capture, although our high follow-up rate (96.7%) minimizes these concerns.
- Single-case observations: Several novel findings represent single-case observations requiring validation in larger series.
- Device evolution: Current FDD technologies may have improved safety profiles compared with devices used during our study period, although basic complication mechanisms remain unchanged.
- Off-label treatment: A proportion of aneurysms are treated in off-label vascular locations, reflecting broadened real-world indications. This may limit the generalizability of our results to strictly on-label use. Recent evidence supports the safety and efficacy of such expanded indications [16].

### Future research directions

This study establishes several important research priorities:

1. Prospective validation of the 18-month safety threshold across contemporary device platforms
2. Mechanistic studies of multiple device complications to optimize complex treatment strategies
3. Investigation of antiplatelet withdrawal phenomena to prove evidence-based discontinuation protocols
4. Research on long-term comparative effectiveness across different FDD platforms via contemporary techniques

## Conclusion

This comprehensive long-term analysis provides definitive evidence that FDD treatment offers excellent durability and safety across multiple device platforms. The critical finding that complications become virtually absent beyond 18 months proves a new paradigm for long-term risk assessment and patient counseling. However, the sevenfold increased risk associated with multiple overlapping devices needs careful treatment planning and enhanced surveillance protocols for complex cases.

These findings significantly advance our understanding of FDD long-term outcomes and provide quantitative evidence to guide contemporary clinical practice. The multidevice platform analysis demonstrated universal safety principles that transcended individual device characteristics, whereas the extended follow-up data offered unprecedented insights into very long-term treatment durability.

For clinical practice, these results support the confident use of FDD technology for proper indications while emphasizing the importance of single-device strategies when feasible. The established 18-month safety threshold provides valuable guidance for patient counseling and surveillance protocol development, contributing to evidence-based FDD treatment paradigms that optimize both safety and efficacy outcomes.

## Data Availability

Data sharing is not applicable to this article as no datasets were generated or analyzed during the current study that are publicly available.

## Abbreviations

FDDs: Flow diverter devices
PED: Pipeline embolization device
mRS: Modified Rankin scale
DSA: Digital subtraction angiography
OKM: O’Kelly–Marotta
CTA: Computed tomography angiography
MRA: Magnetic resonance angiography
TIA: Transient ischemic attack
ICA: Internal carotid artery
MCA: Middle cerebral artery
PCOM: Posterior communicating artery
ACA: Anterior cerebral artery
ISS: Intrastent stenosis
EVT: Endovascular treatment
ISAT: International Subarachnoid Aneurysm Trial
